# Using machine learning techniques to predict the risk of osteoporosis based on nationwide chronic disease data

**DOI:** 10.1038/s41598-024-56114-1

**Published:** 2024-03-04

**Authors:** Jun-Bo Tu, Wei-Jie Liao, Wen-Cai Liu, Xing-Hua Gao

**Affiliations:** 1 Department of Orthopaedics, Xinfeng County People’s Hospital, Jiangxi, 341600 Xinfeng China; 2 Department of ICU, GanZhou People’s Hospital, GanZhou, 341000 Jiangxi China; 3 Department of Orthopaedics, Shanghai Sixth People’s Hospital Affiliated to Shanghai Jiao Tong University School of Medicine, 600 Yishan Road, Shanghai, 200233 China; 4 Department of Orthopaedics, Guangzhou First People’s Hospital, South China University of Technology, Guangzhou, 510180 China

**Keywords:** Osteoporosis, Machine learning, Predict, Stacker, Chronic disease, Risk factors, Endocrine system and metabolic diseases, Metabolic disorders, Trauma

## Abstract

Osteoporosis is a major public health concern that significantly increases the risk of fractures. The aim of this study was to develop a Machine Learning based predictive model to screen individuals at high risk of osteoporosis based on chronic disease data, thus facilitating early detection and personalized management. A total of 10,000 complete patient records of primary healthcare data in the German Disease Analyzer database (IMS HEALTH) were included, of which 1293 diagnosed with osteoporosis and 8707 without the condition. The demographic characteristics and chronic disease data, including age, gender, lipid disorder, cancer, COPD, hypertension, heart failure, CHD, diabetes, chronic kidney disease, and stroke were collected from electronic health records. Ten different machine learning algorithms were employed to construct the predictive mode. The performance of the model was further validated and the relative importance of features in the model was analyzed. Out of the ten machine learning algorithms, the Stacker model based on Logistic Regression, AdaBoost Classifier, and Gradient Boosting Classifier demonstrated superior performance. The Stacker model demonstrated excellent performance through ten-fold cross-validation on the training set and ROC curve analysis on the test set. The confusion matrix, lift curve and calibration curves indicated that the Stacker model had optimal clinical utility. Further analysis on feature importance highlighted age, gender, lipid metabolism disorders, cancer, and COPD as the top five influential variables. In this study, a predictive model for osteoporosis based on chronic disease data was developed using machine learning. The model shows great potential in early detection and risk stratification of osteoporosis, ultimately facilitating personalized prevention and management strategies.

## Introduction

Osteoporosis, a skeletal disorder characterized by compromised bone strength and increased risk of fractures, poses a significant public health challenge worldwide^1,2^. The adverse outcomes of osteoporosis such as hip and vertebral fractures are associated with substantial morbidity, mortality, and economic costs^3^. Unfortunately, osteoporosis often remains undiagnosed and untreated until the occurrence of a debilitating fracture^2^, hence proactive identification of high-risk individuals is a critical step in mitigating the disease burden.

Many studies have recognized common risk factors for osteoporosis, including aging, female gender, family history, low body mass index, and certain lifestyle factors (smoking, excessive alcohol, lack of physical activity)^1,2^. Moreover, several chronic diseases such as rheumatoid arthritis, diabetes, and kidney diseases have been associated with an increased risk of osteoporosis^4^. Nevertheless, the complexity of interactions between these risk factors makes it challenging to accurately predict osteoporosis risk in the individual patient using traditional statistical methods^5^.

Recent advances in artificial intelligence (AI) and machine learning (ML) technologies offer promising opportunities for enhancing risk prediction^6–8^. ML algorithms can handle high-dimensional data and capture complex, nonlinear relationships between predictors, making them particularly suited for developing prediction models based on multifactorial disease data^9,10^. Indeed, ML has shown potential in various healthcare applications, including disease diagnosis and prognosis, treatment response prediction, and patient stratification^11,12^.

However, the development and validation of a ML predictive model for osteoporosis risk, particularly one that is based on chronic disease data, remains unexplored^13^. In present study, we aim to develop a ML-based predictive model for estimating osteoporosis risk using a comprehensive set of chronic disease data. Our model is expected to assist community healthcare workers in screening individuals at high-risk of osteoporosis during health follow-ups using simple indicators, thereby enabling early intervention and preventive measures in high-risk individuals. Ultimately, the results of this study have the potential to contribute to the reduction in fracture incidence, improvement in patient outcomes, and alleviation of the healthcare burden associated with osteoporosis.

Through this research, we aim to construct a predictive model in osteoporosis risk prediction, where machine learning and big data are leveraged to deliver personalized risk assessment and preventive care^6^. This is expected to provide a reference for the adoption and integration of ML technologies in bone health management, and potentially, in the broader context of chronic disease prevention and management.

## Materials and methods

### Study design and data source

This study was designed to develop and validate a machine learning predictive model for the risk of osteoporosis based on a nationwide chronic disease data in Germany. The data used in this study were obtained from 10,000 complete records of open-source primary healthcare data in the German Disease Analyzer database (IMS HEALTH)^14^. This open-source data considered ten different chronic diseases (CDs) based on primary healthcare diagnoses (ICD-10 codes): Hypertension (I10), Lipid metabolism disorders (E78), Diabetes (E10-E14), Coronary heart disease (I20-I25), Cancer (C00-97), Chronic obstructive pulmonary disease (J44), Heart failure (I50), Stroke (I63, I64, G45), Osteoporosis (M80, M81), and Chronic kidney disease (N18, N19).

### Data preparation

Patient data were randomly split into a training set and a test set in a ratio of 7:3 using a stratified random sampling method implemented in Python (version 3.9). This approach ensured that the distribution of osteoporosis cases was similar in both datasets. Label encoding methods were applied to process categorical variables such as smoking and diabetes status.

To address the imbalance of data distribution, the random oversampling method was applied. This method involved duplicating the minority class instances to balance the dataset, improving the model's ability to learn from the underrepresented class^15^.

### Feature selection

In the study, a comprehensive feature screening process was employed to identify the predictors for osteoporosis prediction. We harnessed the power of nine distinct machine learning algorithms to ensure a robust and exhaustive feature elimination process. The selected algorithms were: Logistic Regression (LR), Support Vector Machine (SVM), Decision Tree Classifier (DT), Extra Trees Classifier (ET), Random Forest Classifier (RF), Extreme Gradient Boosting (XGBoost), Light Gradient Boosting Machine (LightGBM), Gradient Boosting Classifier (GBC), and Ada Boost Classifier (ADA). To systematically identify and retain the most informative variables, each of these algorithms was subjected to a recursive feature elimination (RFE) procedure. This method facilitates the optimization of model performance by iteratively removing the least important features based on their predictive power^16^.

### Model development

We employed ten different machine learning algorithms to build predictive models. These algorithms, implemented using scikit-learn, xgboost, and lightgbm modules in Python, included: Logistic Regression (LR), K Neighbors Classifier (KNN), Decision Tree Classifier (DT), Extra Trees Classifier (ET), Random Forest Classifier (RF), Extreme Gradient Boosting (XGBoost), Light Gradient Boosting Machine (Lightgbm), naïve Bayes (NB), Gradient Boosting Classifier (GBC), and Ada Boost Classifier (ADA)^17^.

These algorithms' performance was initially evaluated without hyper-parameter optimization by calculating the area under the receiver operating characteristic curve (AUC-ROC). The top three algorithms were then selected for further refinement. Hyper-parameters of these algorithms were optimized using the randomized search method.

The optimal predictive model turned out to be a stacked ensemble model, utilizing the strengths of LR, ADA, and GBC algorithms. The stacker model was developed through a two-step process. In the first layer, individual models (LR, ADA, and GBC) were trained separately on the training dataset. The predictions from these models were then used as input for the second layer to generate a final prediction.

### Model validation

The best-performing model was validated both internally and externally. For the determination of appropriate cut-off values, the Youden index was utilized, which maximizes the sum of sensitivity and specificity. The external validation was performed using cumulative lift measures, assessing the ratio of the model's prediction capability compared to a random selection. A confusion matrix was used to intuitively represent prediction performance and the discrepancy between the model prediction result and the actual situation.

The performance metrics used for model evaluation included accuracy, sensitivity, specificity, and AUC-ROC. The calibration of the models was assessed by comparing the predicted probabilities with the actual outcomes.

### Statistical analysis

Statistical analyses were conducted using Python (version 3.9, Python Software Foundation). Categorical variables were represented as frequencies or proportions and compared using the chi-square test or Fisher’s exact test. The Kolmogorov–Smirnov-Lilliefors (K-S-L) test was utilized to check the normality of continuous data. Non-normally distributed variables were evaluated using the Wilcoxon rank-sum test and displayed as median, first quartile (Q1), and third quartile (Q3). Differences were deemed significant if *P* < 0.05.

### Feature importance assessment

Shapley Additive Explanations (SHAP) values were used to assess the impact and importance of each input variable on the model's output^18^. SHAP is a game-theoretic method to interpret the output of any machine learning model. It uses classical Shapley values from cooperative game theory, extending them to optimally attribute credit to local explanations. This approach provided insight into which factors were most influential in the prediction of osteoporosis risk.

### Ethics approval

This study was approved by the Ethics Committee of Guangzhou First People's Hospital.

## Results

### Baseline data assessment

Utilizing the open-source primary healthcare dataset provided by IMS HEALTH, this study encompassed a cohort of 10,000 patients. Of these, 8707 (87.07%) presented with osteoporosis, whereas 1293 (12.93%) did not. Comprehensive patient characteristics can be found in Table 1. The cohort was randomly stratified into a training set (n = 7000) and a test set (n = 3000), following a 7:3 distribution. The variable distributions between these two subsets exhibited no statistically significant disparities, as outlined in Table 2.

**Table 1:** Baseline characteristics of study population.

Variables		Overall	No	Yes	*P*-value
N		10,000	8707	1293	
Male gender, n (%)	No	5817 (58.2)	4694 (53.9)	1123 (86.9)	< 0.001
	Yes	4183 (41.8)	4013 (46.1)	170 (13.1)	
Age, median [Q1,Q3]		76.0 [71.0,82.0]	76.0 [71.0,81.0]	79.0 [74.0,85.0]	< 0.001
Hypertension, n (%)	No	3288 (32.9)	3010 (34.6)	278 (21.5)	< 0.001
	Yes	6712 (67.1)	5697 (65.4)	1015 (78.5)	
CHD, n (%)	No	7426 (74.3)	6570 (75.5)	856 (66.2)	< 0.001
	Yes	2574 (25.7)	2137 (24.5)	437 (33.8)	
Lipid disorder, n (%)	No	5880 (58.8)	5247 (60.3)	633 (49.0)	< 0.001
	Yes	4120 (41.2)	3460 (39.7)	660 (51.0)	
Stroke, n (%)	No	9373 (93.7)	8187 (94.0)	1186 (91.7)	0.002
	Yes	627 (6.3)	520 (6.0)	107 (8.3)	
Heart failure, n (%)	No	8431 (84.3)	7464 (85.7)	967 (74.8)	< 0.001
	Yes	1569 (15.7)	1243 (14.3)	326 (25.2)	
Cancer, n (%)	No	8287 (82.9)	7282 (83.6)	1005 (77.7)	< 0.001
	Yes	1713 (17.1)	1425 (16.4)	288 (22.3)	
Diabetes, n (%)	No	6856 (68.6)	5987 (68.8)	869 (67.2)	0.276
	Yes	3144 (31.4)	2720 (31.2)	424 (32.8)	
COPD, n (%)	No	8711 (87.1)	7684 (88.3)	1027 (79.4)	< 0.001
	Yes	1289 (12.9)	1023 (11.7)	266 (20.6)	
Chronic kidney disease, n (%)	No	8699 (87.0)	7629 (87.6)	1070 (82.8)	< 0.001
	Yes	1301 (13.0)	1078 (12.4)	223 (17.2)	

First quartile (Q1), third quartile (Q3).

**Table 2:** Baseline characteristics of training set and test set.

Variables		Overall	Train	Test	*P*-value
N		10,000	7000	3000	
Osteoporosis, n (%)	No	8707 (87.1)	6095 (87.1)	2612 (87.1)	1.000
	Yes	1293 (12.9)	905 (12.9)	388 (12.9)	
Male gender, n (%)	No	5817 (58.2)	4071 (58.2)	1746 (58.2)	0.986
	Yes	4183 (41.8)	2929 (41.8)	1254 (41.8)	
Age, median [Q1,Q3]		76.0 [71.0,82.0]	76.0 [71.0,82.0]	76.0 [71.0,82.0]	0.326
Hypertension, n (%)	No	3288 (32.9)	2290 (32.7)	998 (33.3)	0.606
	Yes	6712 (67.1)	4710 (67.3)	2002 (66.7)	
CHD, n (%)	No	7426 (74.3)	5191 (74.2)	2235 (74.5)	0.738
	Yes	2574 (25.7)	1809 (25.8)	765 (25.5)	
Lipid disorder, n (%)	No	5880 (58.8)	4116 (58.8)	1764 (58.8)	1.000
	Yes	4120 (41.2)	2884 (41.2)	1236 (41.2)	
Stroke, n (%)	No	9373 (93.7)	6575 (93.9)	2798 (93.3)	0.228
	Yes	627 (6.3)	425 (6.1)	202 (6.7)	
Heart failure, n (%)	No	8431 (84.3)	5909 (84.4)	2522 (84.1)	0.683
	Yes	1569 (15.7)	1091 (15.6)	478 (15.9)	
Cancer, n (%)	No	8287 (82.9)	5783 (82.6)	2504 (83.5)	0.314
	Yes	1713 (17.1)	1217 (17.4)	496 (16.5)	
Diabetes, n (%)	No	6856 (68.6)	4819 (68.8)	2037 (67.9)	0.364
	Yes	3144 (31.4)	2181 (31.2)	963 (32.1)	
COPD, n (%)	No	8711 (87.1)	6107 (87.2)	2604 (86.8)	0.567
	Yes	1289 (12.9)	893 (12.8)	396 (13.2)	
Chronic kidney disease, n (%)	No	8699 (87.0)	6110 (87.3)	2589 (86.3)	0.190
	Yes	1301 (13.0)	890 (12.7)	411 (13.7)	

First quartile (Q1), third quartile (Q3).

### Candidate features and algorithms screening

**Figure 1:**
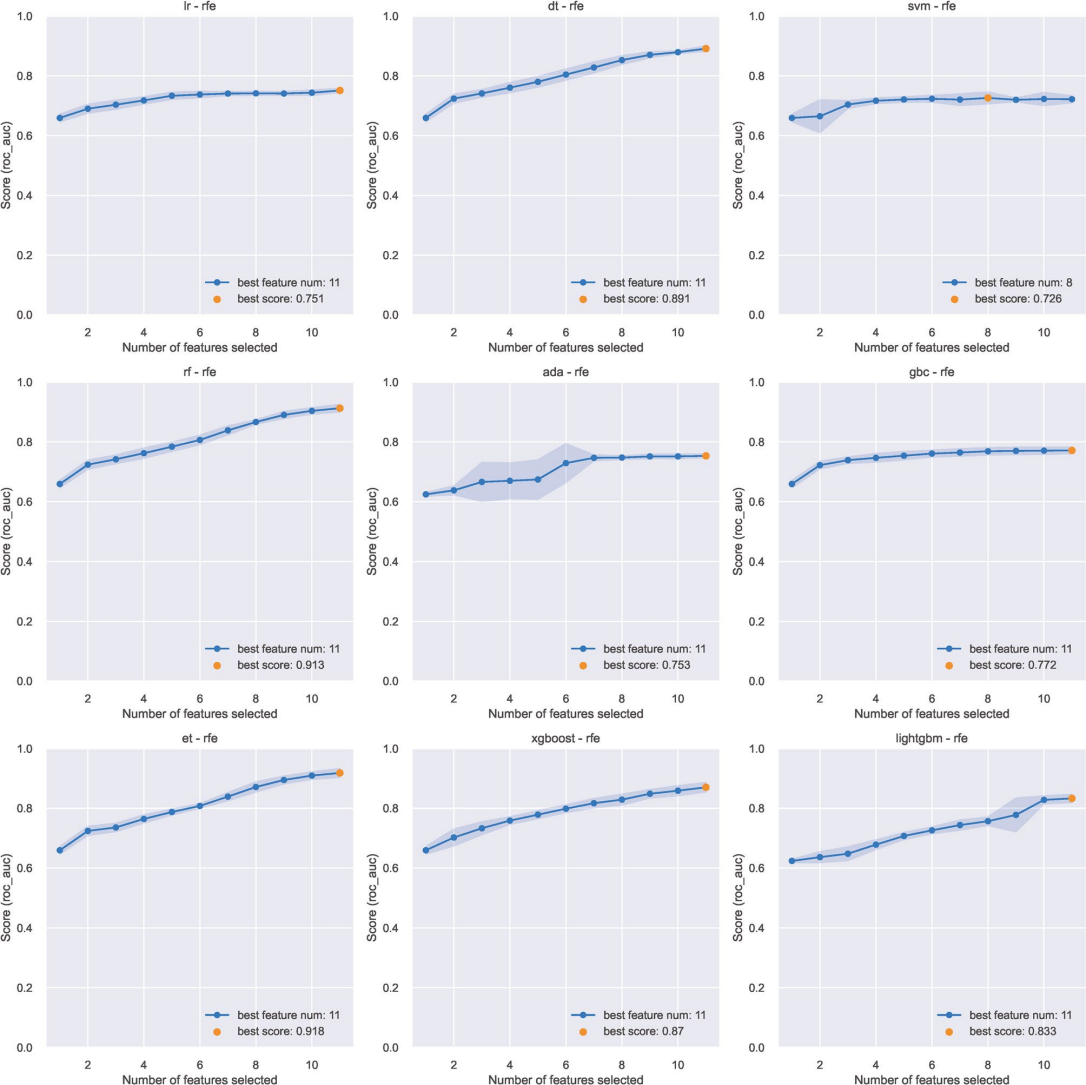
Results of nine different algorithms using recursive feature elimination (RFE) procedure for feature selection. Logistic Regression (LR), Support Vector Machine (SVM), Decision Tree Classifier (DT), Extra Trees Classifier (ET), Random Forest Classifier (RF), Extreme Gradient Boosting (XGBoost), Light Gradient Boosting Machine (LightGBM), Gradient Boosting Classifier (GBC), and Ada Boost Classifier (ADA).

**Figure 2:**
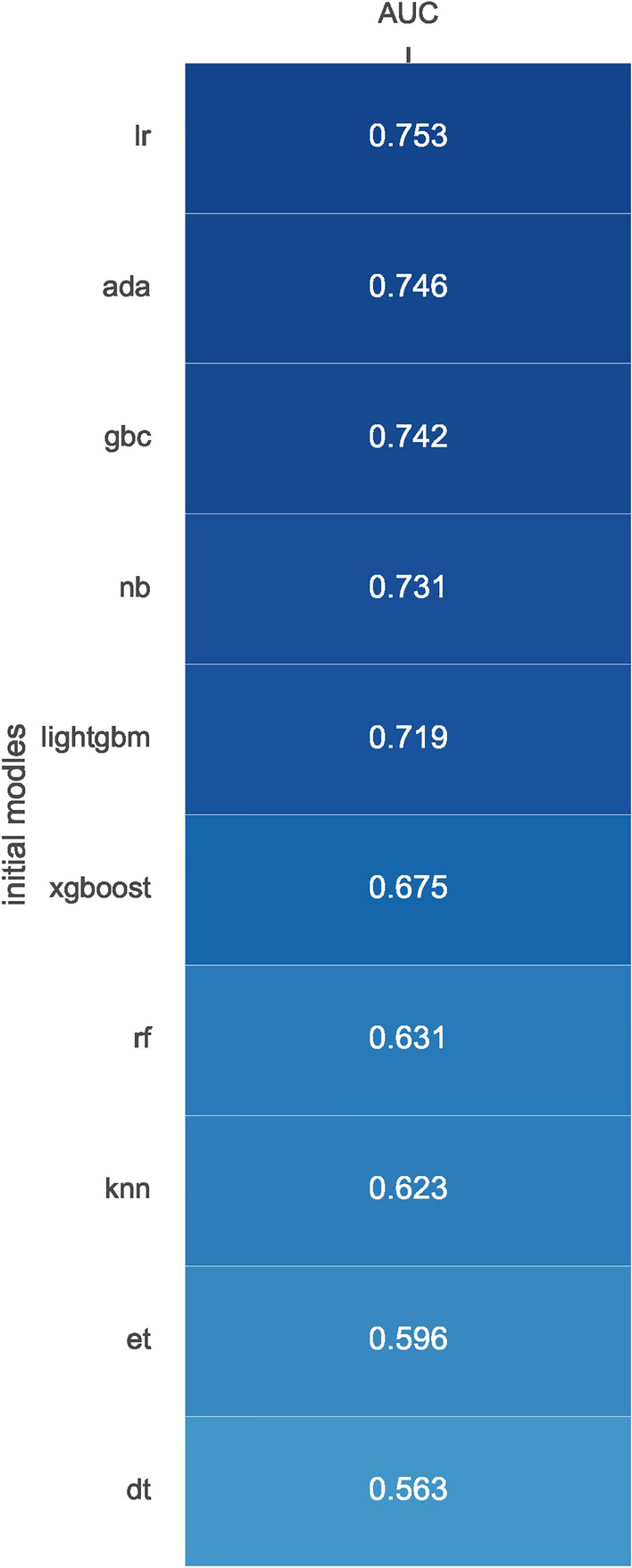
Performance of ten different models in internal validation without initial parameters. Area under receiver operating characteristic curve (AUC); Logistic Regression (LR), K Neighbors Classifier (KNN), Decision Tree Classifier (DT), Extra Trees Classifier (ET), Random Forest Classifier (RF), Extreme Gradient Boosting (XGBoost), Light Gradient Boosting Machine (Lightgbm), naïve Bayes (NB), Gradient Boosting Classifier (GBC), and Ada Boost Classifier (ADA).

A subset of 7000 samples was randomly delineated for the training of models. Within this subset, 6,095 (87.1%) were diagnosed with osteoporosis, contrasting with 905 (12.9%) that were not (Table 2). Referring to the Recursive Feature Elimination (RFE) outcomes across nine distinct algorithms, as visualized in Fig. 1, the data from eight algorithms indicated optimal predictive performance upon inclusion of all eleven predictor variables. Consequently, all features were employed in the construction of predictive models for the study. The initial performances of these ten models in terms of prediction are delineated in Fig. 2. At the preliminary stage of algorithmic assessment, the Area Under the Curve (AUC) was designated as the pivotal metric for performance evaluation. The three superior-performing algorithms earmarked for further exploration were LR (AUC: 0.753), ADA (AUC: 0.746), and GBC (AUC: 0.742), as depicted in Fig. 2.

### Model development and selection

Subsequent to preliminary assessment, the three superior-performing algorithms, namely LR, ADA, and GBC, were earmarked for in-depth refinement. Hyperparameters for these algorithms underwent optimization via the randomized search methodology in tandem with tenfold cross-validation. Then these optimal predictive models turned out to be a stacked ensemble (stacker) model, which synergistically leveraged the robustness of the LR, ADA, and GBC algorithms. This ensemble was constructed via a biphasic approach: the initial phase involved independent training of LR, ADA, and GBC models on the designated training dataset, with their resultant predictions feeding into the second phase to derive a consolidated forecast.

**Figure 3:**
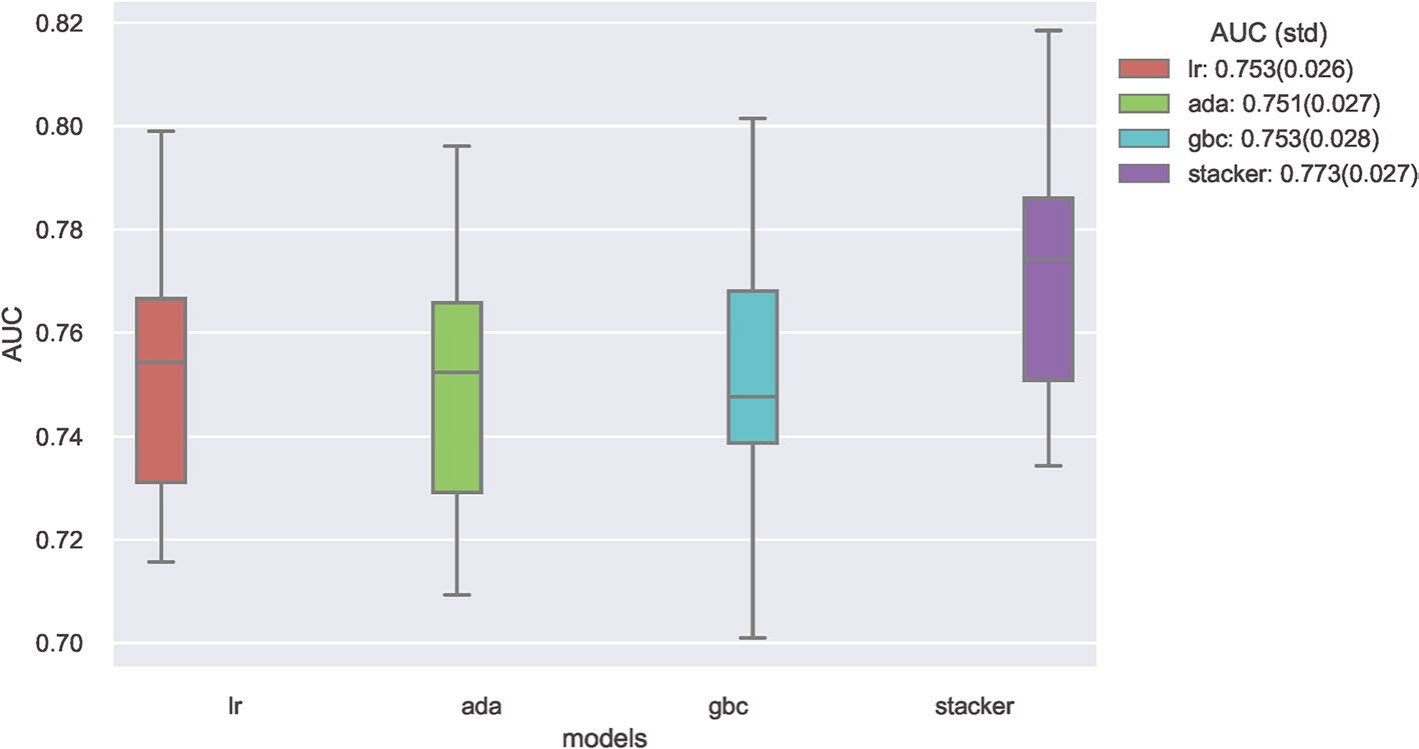
Ten-fold cross-validation results of different machine learning models.

**Figure 4:**
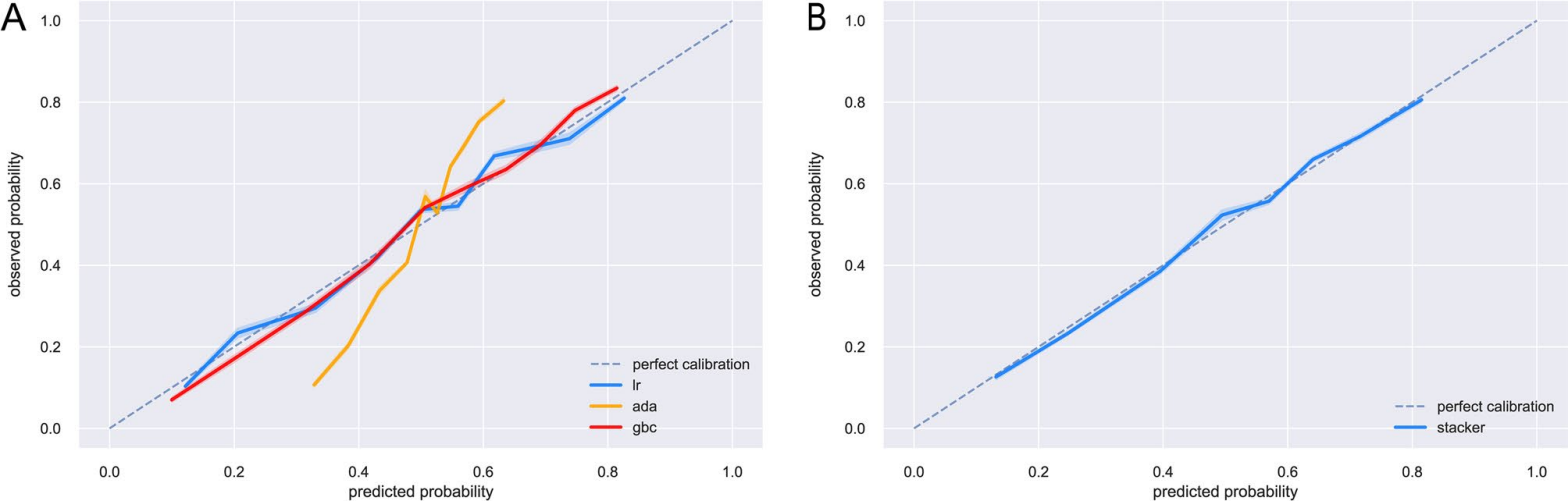
**(A**) The calibration curves of the three models. (**B**) The calibration curves of the stacker model. The diagonal dotted line represents an ideal model and the solid line represents the performance of the model, while the model closer fit to the diagonal dotted line represents a better calibration.

To ascertain the reliability of the devised machine learning constructs, their performance was benchmarked using tenfold cross-validation on the training set, with outcomes delineated in Fig. 3. It was discernible that the stacker model (AUC: 0.773, std: 0.027) showcased enhanced predictive prowess in comparison to standalone LR (AUC: 0.753, std: 0.026), ADA (AUC: 0.751, std: 0.027), and GBC (AUC: 0.753, std: 0.028) models during internal validation. The calibration curves, presented in Fig. 4, furnish insights into model calibration, epitomizing the congruence between predicted osteoporosis risks and the empirically observed outcomes. And the calibration curve associated with the stacker model evinced a good agreement between predictive and observational data.

### Model performance and feature importance

**Figure 5:**
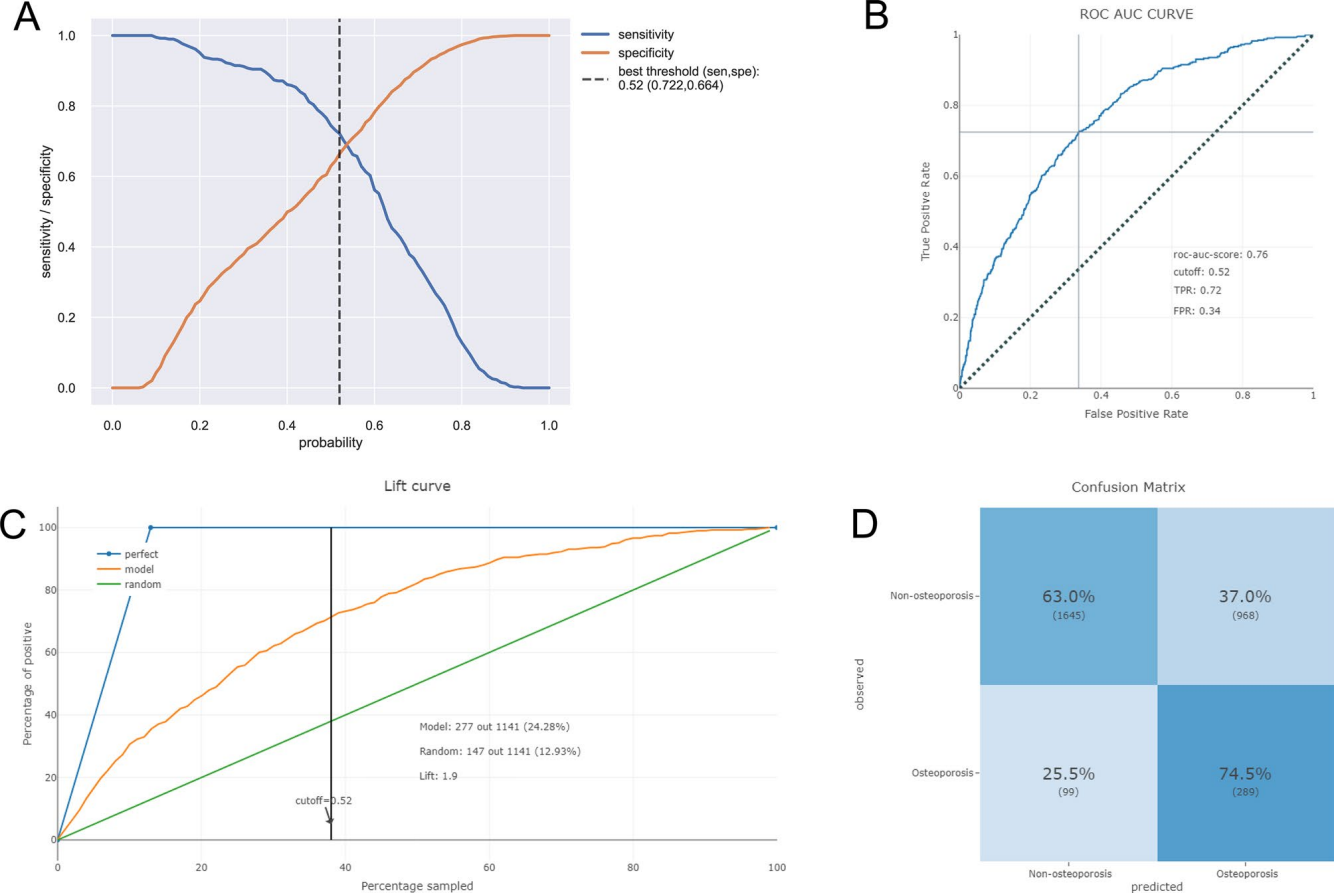
(**A**) Sensitivity and specificity versus cut-off probability plot of the stacker model. Decreasing sensitivity and increasing specificity are shown for increasing probability thresholds for osteoporosis. (**B**) The ROC curves of the stacker model in test set. (**C**) The cumulative lift curve of the stacker model in test set. (**D**) The confusion matrix of the stacker model in the test set.

**Figure 6:**
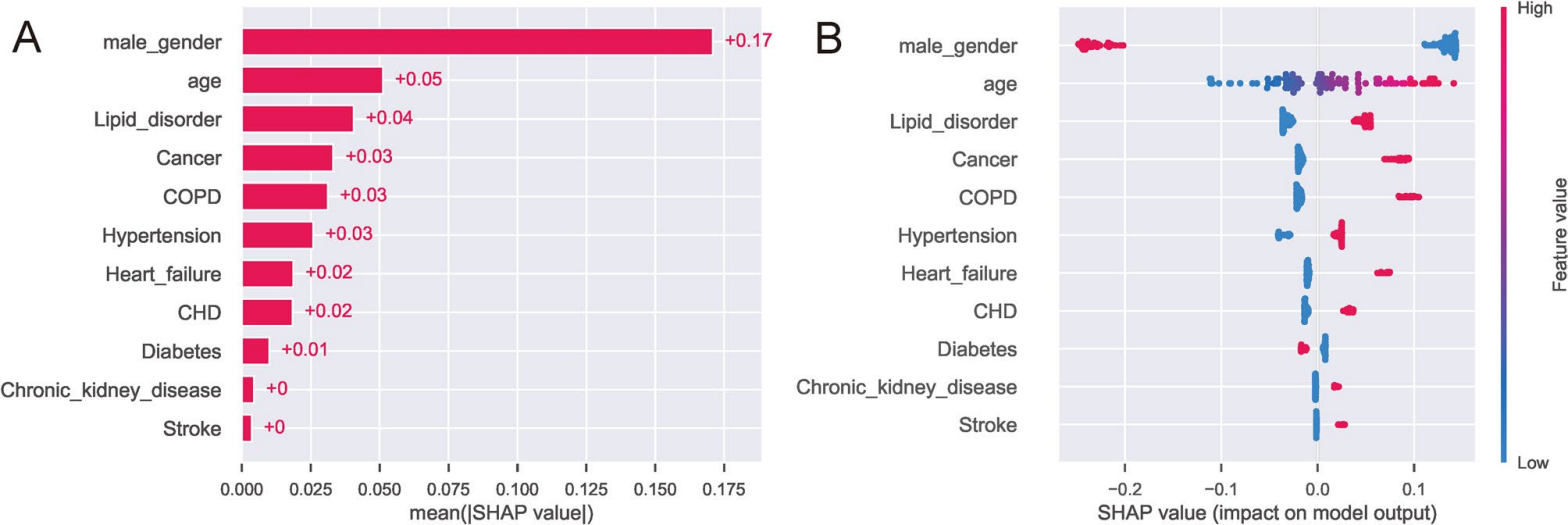
(**A**) Ranking of feature importance of the stacker model based on SHAP values. (**B**) Distribution of the impact of each feature on the output of the stacker model estimated using the SHAP values. The plot sorts features by the sum of SHAP value magnitudes over all samples and shows the order of feature importance. This figure described data from the test cohort, with each point representing one patient. The color represents the feature value (red high, blue low). The x axis measures the impact on the model output (right positive, left negative). A positive value indicate a Osteoporosis risk and a negative value indicate a good outcome.

As delineated in Fig. 5A, with the increasing probability threshold of osteoporosis, there is a decline in sensitivity and an enhancement in specificity. Utilizing the Youden index, an optimal threshold probability of 0.52 was ascertained for the stacker model, yielding sensitivity and specificity metrics of 0.722 and 0.664, respectively. The model's predictive capacity is further illustrated by the cumulative lift in Fig. 5C. This metric reflects the stacker model's ability to identify osteoporosis cases relative to a given sample size when compared to a random selection. In essence, it offered a comparative ratio of patients diagnosed with osteoporosis against those undiagnosed. This was instrumental in contrasting the stacker model's performance against an idealized model (one that predicts osteoporosis flawlessly) and a model based on sheer randomness. With the threshold set at 0.52, the stacker model attained a lift value of 1.9. The ROC curve for the stacker model, applied to the test dataset, was illustrated in Fig. 5B, signaling robust predictive efficacy with an AUC of 0.76. The model's predictive prowess is further underscored by the confusion matrix, as depicted in Fig. 5D. Delving into the feature importance within the stacker model, SHAP values were computed, as visualized in Fig. 6. As shown, age, gender, lipid metabolism disorders, cancer, and COPD as the top five important features for distinguishing the osteoporosis.

## Discussion

Osteoporosis, often termed the "silent disease", is a prevalent condition that reduces bone density, predisposing individuals to increased fracture risk. Notably, the absence of overt symptoms until a fracture occurs underscores the urgency for early detection and preventive strategies^19^. Fractures, particularly hip fractures, associated with osteoporosis, often result in substantial morbidity, increased mortality, and significant health-care costs^20^. The societal and economic implications of osteoporosis-related fractures make predicting the disease an imperative not just from a clinical perspective but also from public health and economic viewpoints^21^.

Early prediction and identification of osteoporosis can pave the way for timely interventions, potentially decelerating or even reversing bone loss. This not only diminishes fracture risk but also bolsters the quality of life for the elderly population, ensuring greater independence and reduced healthcare expenditure^22^. Interventions, which range from lifestyle modifications to pharmaceutical therapies, have shown to be considerably more effective when osteoporosis is identified at nascent stages^23^.

The current research serves as a testament to the potential of machine learning in advancing osteoporosis prediction, highlighting a novel approach that melds the power of various predictive algorithms^24^. Existing methodologies primarily depend on bone mineral density (BMD) tests using DXA scans^25^. Although effective, these tests are not ubiquitously accessible, can be cost-prohibitive, and often are conducted when clinical symptoms manifest, potentially delaying timely intervention.

In many practical scenarios, especially in resource-limited settings like communities, it might be challenging or cost-prohibitive to obtain comprehensive lifestyle data, laboratory test, or advanced imaging results. Thus, building predictive models using data that can be extracted from primary healthcare records or community surveys offers a promising approach for early screening and detection of osteoporosis in these settings. Cheng Li^26^ and colleagues successfully predicted the risk of rotator cuff tears in hospital outpatients using simple questionnaire data and physical examination findings with machine learning techniques. Similarly, Limin Wang et al.^27^ used health questionnaire indicators and regression algorithms to make accurate predictions for symptomatic knee osteoarthritis. By identifying high-risk patients through simple indicators and recommending further precise medical examinations for them, this approach can help reduce unnecessary medical tests and save on healthcare costs.

This study, by capitalizing on nationwide primary healthcare data from Germany, offers a non-invasive and efficient means to predict osteoporosis risk based on health indicators and chronic conditions. The broad inclusion of patients spanning diverse health backgrounds ensures the model's generalizability and applicability in real-world settings. Our aim is to develop a preclinical model that could contribute to early warning and early detection and diagnosis for high-risk populations. In this study, we did not include medical laboratory test indicators and omics data as predictive factors. While this may reduce the model's performance, it also has the advantage of reducing the complexity of the model and enhancing its practicality. Innovation in the field of machine learning does not always mean using the most advanced algorithms or complex feature engineering^28^. Sometimes, simplifying the development of models to improve their universality and usability represents a significant form of innovation. Simple models are easier for other researchers to replicate and validate and are more feasible to implement in real-world settings. Our study results show that the model we developed has an AUC of 0.76, indicating good predictive performance.

The choice of algorithms in the present study was pivotal in ensuring robust prediction performance. The preliminary selection included a range of algorithms, out of which LR, ADA, and GBC emerged as the front-runners in terms of the AUC metric. Previous research in medical diagnostics has emphasized the importance of the AUC as an indicative measure of the model's capability to discriminate between positive and negative instances^29^. Although the research by Meng, Y., et al.^30^ suggests that sequential models, such as GRU or LSTM, may outperform non-sequential models like LR or XGB, the advantages of these models may not be fully leveraged in the context of cross-sectional data alone. In this study, considering the characteristics of the dataset used for modeling, non-sequential models were adopted as the final predictive models, which also achieved good predictive performance. Our findings are congruent with recent literature suggesting the promise of these algorithms in health-related prediction tasks^31–33^.

Ensemble methods have consistently demonstrated their mettle in improving prediction accuracy by combining the strengths of multiple models and ameliorating individual model limitations^34^. The use of a stacked ensemble in our study—a model synergizing the robustness of LR, ADA, and GBC—substantially augmented the AUC during internal validation. This approach capitalizes on the distinct decision boundaries offered by each algorithm, thus providing a holistic, comprehensive prediction. This approach offers higher predictive accuracy over individual models, a finding in alignment with contemporary studies on ensemble methods^35^.

The optimal threshold probability of 0.52 derived from the Youden index underscores the balanced consideration of both sensitivity (true positive rate) and specificity (true negative rate) in the study. This ensures not only the correct identification of actual osteoporosis cases but also minimizes false alarms, which can be critical in clinical applications to avoid overdiagnosis and unnecessary interventions. The achieved lift value of 1.9 for the stacker model accentuates its ability to effectively identify osteoporosis cases compared to random selection, validating its clinical utility.

Furthermore, the comprehensive feature selection process and rigorous validation reaffirm the model's robustness and reliability. The application of SHAP values for feature importance not only fosters transparency in machine learning predictions but also offers clinical insights, helping healthcare practitioners understand and prioritize risk factors^36^.

The SHAP values, an advanced tool for model interpretability, were instrumental in determining the salience of each feature within our predictive framework. Age and gender emerged as the most paramount factors, a finding that resonates with the broader osteoporosis literature. The long-established relationship between advancing age and decreased bone density makes age a pivotal predictor for osteoporosis risk^37^. Gender-specific differences, especially post-menopausal changes in women, exacerbate the risk of osteoporosis, emphasizing its importance in our model^38^.

The significance of lipid metabolism disorders in predicting osteoporosis in our model presented intriguing insights. Recent studies have begun to identify a potential association between dyslipidemia and bone mineral density (BMD) alterations^39,40^. Lipids play a role in bone metabolism, and aberrations in lipid profiles may adversely affect bone health. Our model's emphasis on cancer as a risk factor underscores the multifaceted relationship between cancer and osteoporosis. Some treatments for cancer, especially those involving hormone therapies, can accelerate bone loss, making patients more susceptible to osteoporosis^41,42^. COPD has also been linked to low bone mineral density and a higher risk of fractures. Pulmonary dysfunction and decreased BMD share underlying inflammatory pathways. The chronic inflammatory state in COPD can disrupt bone metabolism, leading to increased osteoporosis risk^43^. Hypertension has been associated with an increased risk of osteoporosis, potentially due to alterations in calcium homeostasis, as well as the effects of antihypertensive medications^44^. Stroke patients also face an increased risk of osteoporosis and fractures, likely due to immobilization and neuronal damage affecting bone metabolism. Similarly, heart failure, CHD, and chronic kidney disease have all been associated with an increased risk of osteoporosis and fractures^45–47^.

The imperative of early osteoporosis prediction has never been clearer. As populations age globally, the public health burden of osteoporotic fractures is poised to rise. Against this backdrop, our study stands as a meaningful stride towards enhancing osteoporosis predictive modalities. Utilizing the open-source primary healthcare dataset from IMS HEALTH, which included records from a large number of patients, we endeavored to develop a machine learning-based predictive model. With further research and validation, we hope the model will assist community healthcare workers in screening patients at high risk of osteoporosis during health follow-ups using simple indicators. Personalized health advice is given to these high-risk patients, and further medical tests such as laboratory tests or radiology are recommended to clarify the diagnosis. This may help to reduce unnecessary medical tests and save healthcare costs while ensuring that the benefits to osteoporosis patients.

Several algorithms were assessed in our endeavor, with the stacked ensemble approach of combining Logistic Regression (LR), Ada Boost Classifier (ADA), and Gradient Boosted Classifier (GBC) emerging as particularly promising. The superiority of this ensemble model underscores the inherent complexities of osteoporosis prediction. It emphasizes that the disease's multifaceted nature may be best captured by drawing from the strengths of multiple algorithms.

## Limitations

However, it is important to acknowledge the limitations of our study. Our reliance on the IMS HEALTH dataset confines our findings to its demographic and geographic specifications. Consequently, the external validity and generalizability to other populations or regions might be limited. The ensemble model, for all its predictive prowess, also adds an element of complexity. Its seamless integration into clinical settings, especially ones with limited resources, could be a challenge. The cross-sectional nature of our dataset provides just a snapshot, whereas osteoporosis's progression warrants a more longitudinal analysis. And for this reason, we did not apply sequential model in our study. In addition, due to the limitations of the information contained in the database, our model did not incorporate factors such as diet, lifestyle, physical activity and genetic predisposition, which reduces the complexity of the model but at the same time has an impact on the performance of the model. In the future, we hope to collect more dimensions of data to conduct more in-depth and robust studies in further research and validation. Moreover, while our model identified several chronic conditions as key predictors of osteoporosis risk, it is important to note that these conditions do not operate in isolation. They often interact with each other and with other factors such as lifestyle and genetics in ways that can either exacerbate or mitigate the risk of osteoporosis. Therefore, a thorough understanding of these interactions and their implications for osteoporosis risk is necessary for the accurate interpretation and application of our model's predictions.

## Conclusion

In conclusion, the study highlighted the potential of using ML techniques for predicting osteoporosis risk based on chronic disease data. The stacker model, incorporating a diverse set of variables related to age, gender, and chronic diseases, demonstrates good predictive performance and offered a tool for individualized osteoporosis risk management and early warning and detection, which could facilitate early interventions and improve patient outcomes.

## Data Availability

The raw data for this paper is all open access and can be accessed from the following link: https://doi.org/10.5061/dryad.qh0h1.
